# Biatrial transeptal atriotomy approach to the mitral valve: V-atriotomy technique

**DOI:** 10.1186/1749-8090-8-S1-P165

**Published:** 2013-09-11

**Authors:** A Kucuker, G Ozerdem, M Hidiroglu, L Cetin, B Kaya, E Sener

**Affiliations:** 1 Department of Cardiovascular Surgery, Izmir Katip Celebi University, Ataturk Training and Research Hospital, Karsiyaka-Izmir, Turkey; 2 Cardiovascular Surgery Department, Kayseri Sevgi Hospita, Kayseri, Turkey

## Background

Mitral valve surgery can sometimes be challenging because of inadequate exposure due to a deep chest or a small left atrium. We report our experience with an alternative surgical approach to the mitral valve through biatrial transeptal incision both to the left and right atrium which we named V-atriotomy.

## Methods

61 patients undergoing valve surgery were managed with V-atriotomy incision because of inadequate exposure of the mitral valve and subvalvular apparatus. 37 patients were male (60.7%) and 24 patients were female (39,3%) with a mean age of 64.7±10,2. Standart left atriotomy incision was made primarily and in cases of bad exposure, caval snares were tightened for total perfusion. Right atriotomy incision was done as well followed by interatrial septum incision. Interatrial septostomy incision and left atriotomy incision were connected at 2 cm medial to inferior vena cava cannula. Suspensory sutures were placed to the free edges of right atriotomy and interatrial septostomy. This excellent visualisation of the mitral valve leaded us to perform valve repair or replacement procedures.

## Results

Mitral valve repair was performed for 32 patients (52,5%) and mitral valve replacement for 29 (47.5%). Mean cardiopulmonary bypass time was 143,62±42,74 and cross clamping time was 95,57±24,67 minutes. Intraaortic balloon counterpulsation was required for 5(8.2%) patients. Postoperative antiarythmic drug was used for 12(19.8%) patients. No patient needed permanent pacemaker.

## Conclusion

Good exposure is mandatory for mitral valve repair since meticulous analysis of the valve and subvalvular apparatus is essential. We suggest that V atriotomy approach is a useful alternative, particularly for re-operations, ischemic mitral disease and at small left atrium size.

